# Visuospatial working memory and obstacle crossing in young and older people

**DOI:** 10.1007/s00221-022-06458-9

**Published:** 2022-09-16

**Authors:** N. C. W. Chu, D. L. Sturnieks, S. R. Lord, J. C. Menant

**Affiliations:** 1 Neuroscience Research Australia, University of New South Wales, Sydney, NSW Australia; 2 School of Medical Sciences, University of New South Wales, Sydney, NSW Australia; 3 School of Population Health, University of New South Wales, Sydney, NSW Australia

**Keywords:** Aged, Obstacle avoidance, Working memory, Secondary task, Visuospatial processing

## Abstract

Obstacle crossing requires visuospatial working memory to guide the trailing leg trajectory when vision in unavailable. Visuospatial working memory, as assessed with neuropsychological tests, declines with age, however, this remains to be investigated functionally in obstacle crossing. There is also evidence that visuospatial encoding during a secondary task interferes with balance control during stepping and walking in older people. Here, we studied the interaction effects of age by delay (study 1) and age by secondary visuospatial task (study 2) conditions on obstacle clearance in a visuospatial working memory -guided obstacle crossing task. Healthy young adults aged 19 to 36 years (*n* = 20 in study 1 and *n* = 17 in study 2) and healthy older adults aged 66 to 83 years (*n* = 29 in study 1 and *n* = 21 in study 2) were instructed to step over an obstacle with their leading leg and straddle it for a delay period before completing the crossing with their trailing leg. In study 1, two obstacle height conditions (12 cm, 18 cm) and two delay durations (20 s, 60 s) were presented in random order. In study 2, participants were required to attend to either no secondary task (control), a visuospatial secondary (star movement) task, or a nonspatial secondary (arithmetic) task, while straddling the obstacle for a delay duration of 20 s, at obstacle heights of 12 cm and 18 cm, randomly presented. Trailing leg kinematics (mean and variability of maximum toe clearance over the obstacle) were determined via motion capture. There were no statistically significant age by delay or age by secondary task interactions. In study 1, toe clearance variability was significantly greater in young adults and increased with increasing delay duration in both groups. In study 2, compared with the control condition, toe clearance variability was significantly greater in the non-spatial secondary task condition but not in the visuospatial condition. Contrary to our hypotheses, these findings suggest that young and older adults alike can store an obstacle representation via visuospatial working memory for durations of at least 60 s and use this information to safely scale their trailing leg over an obstacle. However, the increase in trailing leg toe clearance variability with delay duration suggests that obstacle representation starts to deteriorate even within the first 20 s regardless of age. The finding that undertaking a concurrent arithmetic task impaired visuospatial working memory-guided obstacle clearance suggests a potential increased risk of tripping during obstacle crossing while dual-tasking in both young and older people.

**Supplementary Information:**

The online version contains supplementary material available at 10.1007/s00221-022-06458-9.

## Introduction

Most falls in older people occur during ambulation and obstacle crossing, accounting for 50–70% and 11% of falls, respectively (Lord et al. 2007; Milat et al. 2009). This is unsurprising as obstacle crossing is a common challenge of ambulation in daily life, such as when avoiding cracks in a footpath or climbing stairs. Maladaptation of gait to an obstacle could cause collision and falling.

Obstacle crossing is a complex goal-directed task requiring higher level cognitive processes, including executive function, attention, visuospatial processing and motor planning (Chen et al. 2017; Mirelman et al. 2017; Mollaei, et al. 2017; Shumway-Cook et al. 2007; Yogev-Seligmann et al. 2008). According to recent studies in quadrupeds and humans, visuospatial working memory (VSWM) is strongly implicated in obstacle crossing; where it refers to the temporary storage of visuospatial information used to guide ongoing behaviour (Baddeley 2003; Pasternak et al. 2005). This form of working memory is particularly important in obstacle crossing, where a stored visuospatial representation of an obstacle is used to guide both legs over the obstacle when direct visualisation of the obstacle becomes unavailable (Yogev-Seligmann et al. 2008; Lajoie et al. 2012; Mohagheghi et al. 2004; Rhea et al. 2011; Shinya et al. 2012).

Studies in cats and horses have demonstrated that quadrupeds can successfully cross an obstacle with their forelegs and straddle it between their forelegs and hindlegs for a delay period, before subsequently crossing the obstacle with their hindlegs (McVea et al. 2006; McVea et al. 2007; Whishaw et al. 2009; Wong et al. 2017). This task relies on VSWM to guide hindleg obstacle crossing, since visualisation of the obstacle is unavailable. In these experiments, precise visuospatial information of the obstacle’s height (demonstrated by scaling their hindlegs over the obstacle) was retained for delay durations of up to 10 min in cats (McVea et al. 2006; McVea et al. 2007) and 15 min in horses (Whishaw et al. 2009). Using a similar obstacle-crossing paradigm with young adults, Lajoie et al. (Lajoie et al. 2012) showed that young adults can likewise retain precise visuospatial information of an obstacle’s height for delay durations of at least 2 min, as demonstrated by appropriate scaling of their trailing leg over the obstacle following the delay duration.

Notably, VSWM has been found to deteriorate with age. This age-related decline has been widely demonstrated using various pen-and-paper tests of VSWM, whereby older adults perform significantly worse than young adults (Mattay et al. 2006; Heinzel et al. 2017; Lubitz et al. 2017; Suzuki, et al. 2018). Furthermore, the accuracy of a VSWM-guided upper limb grasping response has also been shown to decline with age (Cheng et al. 2012). However, the decline of functional VSWM with age remains poorly understood and has not been investigated in the context of lower limb obstacle crossing to date.

Moreover, dual tasking is a common requirement of daily life, for example having a conversation while walking. Previous dual-task studies have demonstrated that undertaking a secondary cognitive task significantly compromises performance in gait and balance tasks, which may increase fall risk (Yogev-Seligmann et al. 2008; Worden et al. 2016; Maylor et al. 1996; Woollacott et al. 2002; Menant, et al. 2014). Those which have focused on obstacle crossing have confirmed the detrimental impact of ageing (Brown et al. 2005; Kim et al. 2007) and of tasks diverting gaze (Worden et al. 2016; Cho et al. 2019) or requiring increasing VS attention (Lo et al. 2015) on safe clearance and/or secondary task performance. However, a comparison of the effects of secondary tasks tapping in different cognitive domains on obstacle crossing performance, in young and older adults, has yet to be investigated.

A dual-task paradigm may prove useful for exploring what type of cognitive secondary task interferes with obstacle representation in VSWM, thereby adversely affecting obstacle crossing in older people. We and others have reported that compared to nonspatial cognitive tasks, visuospatial cognitive tasks are more disruptive to balance control during standing, stepping and walking, possibly due to competition for common networks for spatial information encoding (Menant, et al. 2014; Sturnieks et al. 2008a; Woollacott et al. 2008; Barra et al. 2006; Kerr et al. 1985; Nadkarni et al. 2010).

To address the above issues, we conducted two complementary studies. Study 1 investigated the effects of age and delay duration on obstacle representation in VSWM, using kinematic data (toe clearance). We hypothesised an age group by delay duration interaction effect on kinematic data, whereby young and older participants would have no significant difference in mean and variability of toe clearance at the shorter delay duration; but at the longer delay duration, older participants would exhibit significantly greater trailing leg toe clearance variability than young participants. This hypothesis was registered prior to data analyses on the Open Science Framework (osf.io/f65b4).

Study 2 investigated the effects of a visuospatial cognitive task versus a nonspatial cognitive task on obstacle representation in VSWM during an obstacle crossing task in young and older adults. We hypothesised that if obstacle crossing is dependent on VSWM: (i) the visuospatial cognitive task would produce a greater increase in intra-subject variability of trailing leg toe clearance during obstacle crossing, compared to the nonspatial cognitive task and the control task; and (ii) this effect would be more pronounced in older adults. We, therefore, hypothesised a dual-task type condition by age group interaction effect on toe clearance variability, which was registered prior to data analyses on the Open Science Framework (osf.io/t3c8e).

## Methods

### Participants

Twenty healthy young adults and 29 healthy older adults participated in study 1, and 17 healthy young adults and 21 healthy older adults participated in study 2. Young participants were recruited from among staff and students of Neuroscience Research Australia (NeuRA), whereas older participants were recruited from the NeuRA volunteer registry and previous research participants who had indicated an interest in future studies at NeuRA (Sturnieks et al. 2019).

The inclusion criteria were a Mini-Mental State Examination (MMSE) (Tombaugh 1992) score of at least 24, independence in activities of daily living, and the ability to ambulate at least 20 m without the use of a mobility aid. The exclusion criteria were medical or psychological conditions that may have precluded safe participation in the study. All participants provided informed written consent as approved by the University of New South Wales Human Research Ethics Committee (HC15580).

### Protocol

**Fig. 1:**
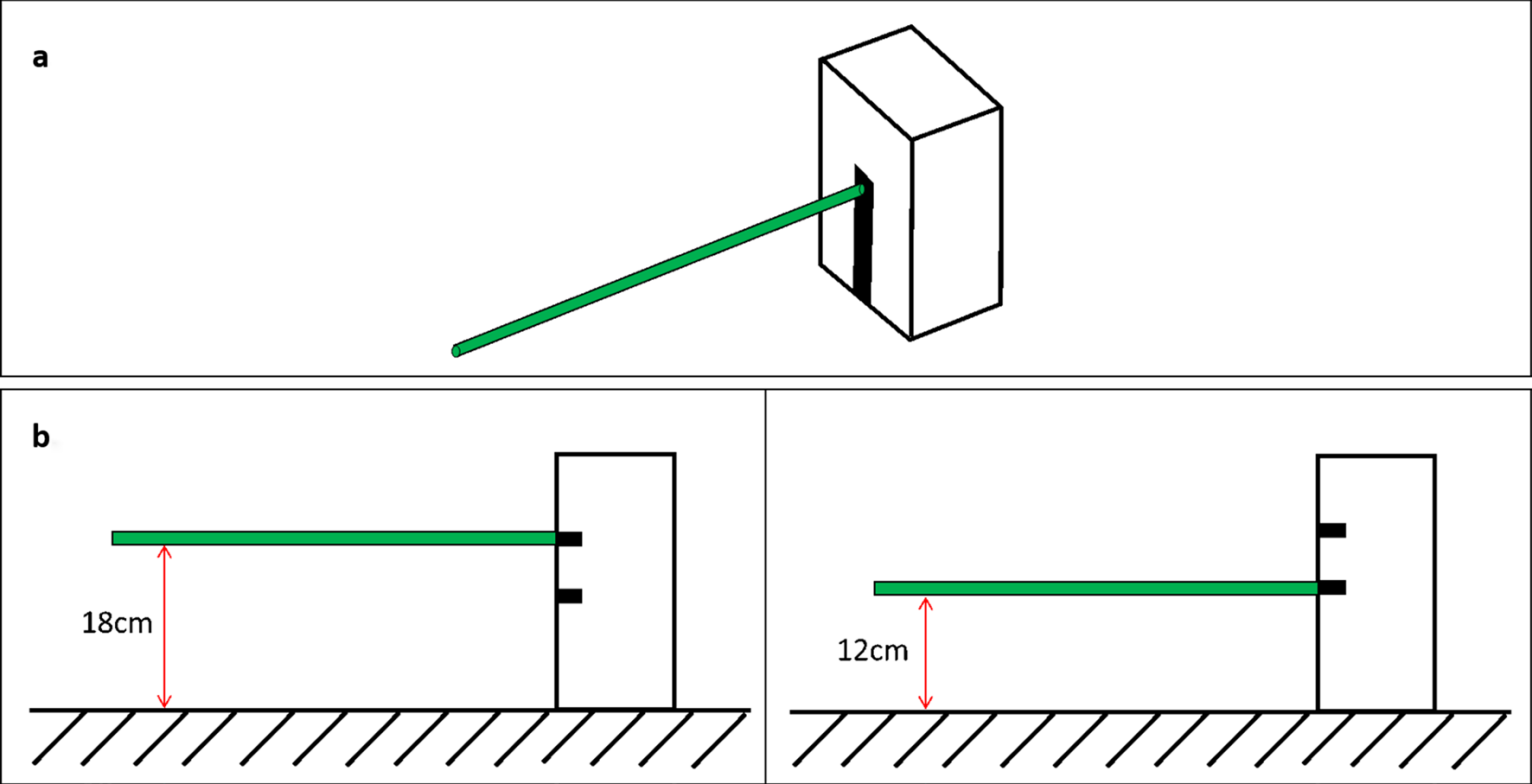
**a** The obstacle (denoted by the green rod) attached at one end to a holder. **b** frontal view of the obstacle (denoted by the green bar) set at each of the two obstacle heights

Participants visited NeuRA on one (single study) or two (both studies) occasions for assessment, first completing a short battery of neuropsychological tests. This study adapted the obstacle-straddling paradigm described by Lajoie and colleagues (Lajoie et al. 2012). Here, the obstacle was a 1 m-long wooden rod of 1 cm diameter painted bright green and attached at one end to a strut (Fig. 1), that could hold the rod at two different heights—18 cm (high) and 12 cm (low) (Fig. 1). The selection of two different obstacle heights (presented in random order) used in both studies was to ensure participants remained attentive to the obstacle height in each trial, and to prevent participants acquiring a learned motor pattern to clear the obstacle. The 18 cm obstacle height was selected to reproduce the standard step height in Australia (Standards Association of Australia 1992), whereas the 12 cm obstacle height was selected to be sufficiently different than the former obstacle height. Participants were provided with Oxford-style standard shoes to wear throughout the experiment and were asked which leg they would choose to stand on one leg, to ascertain leg dominance.

#### Descriptive tests

The mini-mental state examination (MMSE) provided a global measure of cognitive function to ascertain fulfilment of the inclusion criteria (Tombaugh 1992). The trail making test (TMT) assessed executive functions of cognitive-motor speed and task-switching ability; and the difference between parts A and B of the test was calculated to provide a measure of executive function (by eliminating the speed and motor elements from the test administration) (Wechsler et al. 1981). The Wechsler Adult Intelligence Scale-III (WAIS-III) Digit Span Backwards subtest assessed verbal working memory (Wechsler et al. 1997); and the visuospatial star movement task assessed visuospatial working memory (Menant et al. 2014; Sturnieks et al. 2008a). Finally, corrected visual acuity was assessed binocularly with a 6 m-logMAR chart.

#### Study 1

In study 1, participants performed a block of six control trials, followed by two blocks of twelve experimental trials each. Participants were given a rest break in between each block. Prior to the control trials, participants performed some practice trials to identify the individual starting position and familiarise themselves with the shoes and the straddling task.

*Control trials* At the beginning of each trial, participants stood at a fixed starting position in front of the obstacle. Upon verbal instruction, participants took one step with the non-dominant leg, then stepped over the obstacle with the dominant (leading) leg before immediately stepping over the obstacle with the non-dominant (trailing) leg and continued walking for a few steps to cross an end line ~ 2 m away.

**Fig. 2:**
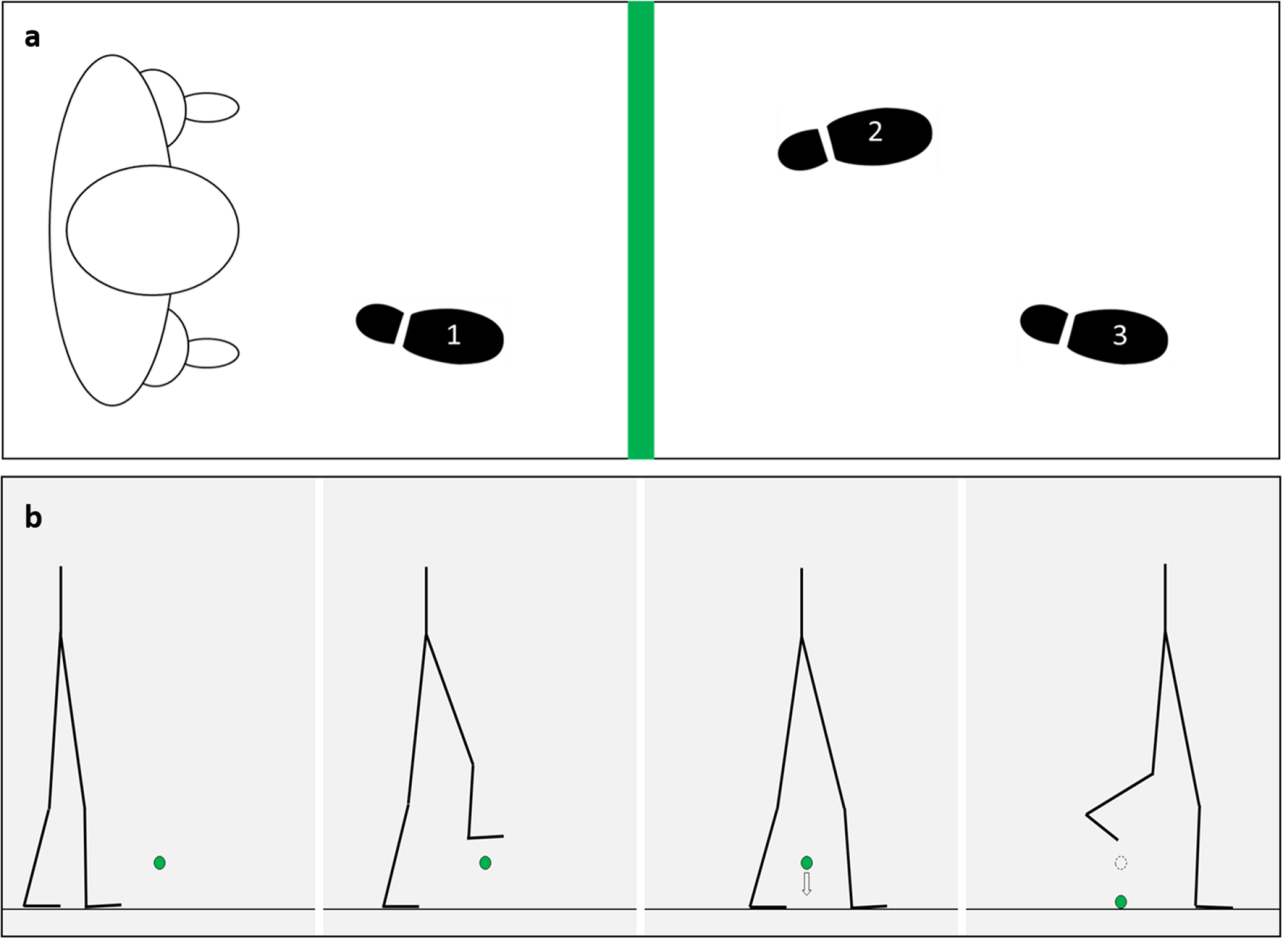
Sequence of steps in the obstacle-straddling paradigm; **a** overhead view of the sequence of foot placements taken by participants to step over the obstacle (green bar denotes the obstacle; footprints denote sequence of foot placements). **b** lateral view of the steps taken by participants to step over the obstacle; first crossing it with the leading leg, then pausing and straddling the obstacle for a delay duration during which the obstacle was lowered to the ground, before subsequently stepping over the obstacle with the trailing leg (green dot denotes the obstacle; arrow denotes the obstacle being lowered to the ground; dotted circle denotes original height of the obstacle before being lowered)

*Experimental trials* At the beginning of each experimental trial, participants stood at a fixed starting position in front of the obstacle. Upon verbal instruction, participants took one step with the non-dominant leg, then stepped over the obstacle with the dominant (leading) leg. At this stage, participants were required to remain straddling the obstacle for one of two delay periods—20 s (short) and 60 s (long). During the delay period, participants were instructed to remain still and refrain from looking down at the obstacle but fix their gaze on a visual target (a black and white cross) placed at eye-level 4 m in front of them. During this period, an examiner (concealed behind a curtain and out of view of the participant) lowered the obstacle to the ground without the participant’s knowledge, to ensure the participant would not contact the obstacle if they underestimated its height. After the delay period, a beep tone signalled for the participant to step over the obstacle with their non-dominant (trailing) leg, without looking down at the obstacle (Fig. 2). The obstacle height and delay duration were presented in a random order across experimental trials.

#### Study 2

In study 2, participants were required to attend to either no secondary task (control), a visuospatial (star movement) secondary task, or a nonspatial (arithmetic) secondary task while straddling the obstacle for a delay duration of 20 s. Participants performed six trials of each condition (three at each height) presented in a block-randomised order. The delay duration of 20 s was selected to minimise fatigue and monotony.

*Experimental set-up* Throughout the delay period, secondary task instructions were delivered continuously to the participants as a voice recording via loudspeaker to ensure uninterrupted engagement with the secondary task; that is, a new question was delivered immediately after participants answered the previous question. During the delay period, the obstacle was also lowered to the ground without the participant’s knowledge; after the delay period, a beep tone signalled for the participant to complete the step over the obstacle without looking down at it (Fig. 2).

*Cognitive secondary tasks* The visuospatial (star movement) task has been previously described (Menant et al. 2014; Sturnieks et al. 2008a), and required participants to envisage four boxes arranged in a 2 × 2 matrix labelled A to D. Participants were asked to mentally visualise a star located in one of the boxes making four movements (comprising up, down, left, right, and diagonal moves), then state the final location of the star. The nonspatial (arithmetic) task required participants to perform additions and subtractions using four single digit numerals, but with a running total that was always > 0. In the control condition, participants were instructed to refrain from mental engagement.

The secondary tasks selected required equivalent verbal responses of mostly one syllable, and both secondary tasks were previously shown to be matched for difficulty (Menant, et al. 2014; Sturnieks et al. 2008a). Thus, any confounding effect of speech or task complexity would be consistent across both secondary task conditions. Participants practised both secondary task conditions to ensure they were proficient in conducting them before commencing experimental trials. The percentage of questions answered correctly were recorded to assess accuracy of secondary task performance.

### Data collection

Kinematic data (toe clearance and maximum toe elevation) were measured using a 7-camera motion capture system (OptiTrack, NaturalPoint, Oregon, USA) at a sampling rate of 100 Hz, using passive retroreflective markers. The toe markers were placed at the same relative location on all shoes, on the upper surface of the shoes, superior to the second metatarsal head.

### Data analysis

Toe clearance (TC) was calculated for the trailing leg, maximum toe elevation over the obstacle was measured for both legs, as well as the intra-subject variability of TC. Toe clearance was defined as the vertical distance between the toe marker and the upper border of the obstacle when the toe marker of the crossing foot was directly over the obstacle (i-e, the toe marker anterior–posterior position was aligned with that of the obstacle). The trailing leg TC was derived as the obstacle was lowered and thus was not in position to require clearing. Toe clearance variability was calculated as the standard deviation of the mean TC. Maximal toe elevation was computed to confirm that participants were scaling their foot trajectory to obstacle height. It was defined as the maximum vertical displacement of the crossing leg toe marker from the ground during the stepping trajectory over the obstacle. The mean trailing leg TC represents the accuracy of obstacle crossing performance, while the intra-subject variability of the trailing leg TC represents the consistency of obstacle crossing performance. These kinematic data were computed and collated for each age group and task condition using a custom-written code in MATLAB (MATLAB R2018, The MathWorks Inc., Natick, Massachusetts, USA). We did not hypothesise a statistically significant effect of obstacle height on trailing leg TC variability; therefore, after pooling the obstacle heights data, the trailing TC variability data were computed from 6 trials for the control condition (zero-delay), 12 trials in each experimental delay condition in Study 1, and 6 trials in each secondary task condition in Study 2 (control, NS and VS).

For study 1, a sample size of 50 (20 young and 30 older adults) was thought to be sufficient to detect a meaningful between-group difference for the main dependent variable (trailing leg obstacle clearance height), i.e. a difference of 25% in foot clearance over the obstacle (Hatton et al. 2013). For study 2, a convenience sample was gathered that reflected the resources available for the study (time, funding and personnel).

### Statistical analysis

Statistical analysis of the collected data was completed using IBM Statistical Package for Social Sciences (SPSS) Statistics Version 23 (IBM Corp., New York, USA). The data were visually examined for outliers and normality of distribution, and skewed data were log transformed. In study 1, a mixed-model repeated measures analysis of variance (ANOVA) was performed on the kinematic variables with delay duration (zero, 20 s, 60 s) and obstacle height (low, high) as within-subject factors, and age group (young, older) as a between-subject factor. The zero-delay condition was included in the analysis to investigate potential deteriorations which might have occurred within the first 20 s period. In study 2, a mixed-model repeated measures ANOVA was performed on the kinematic variables with secondary task condition (control, nonspatial, visuospatial) and obstacle height (low, high) as within-subject factors, and age group (young, older) as a between-subject factor. In both studies, where a significant interaction effect or main effect was identified, post hoc tests using the Least Significant Difference method were performed. The null hypothesis was rejected if *p* < 0.05.

## Results

### Description of the sample

Table 1 presents basic demographic, anthropometric, and cognitive test performance information for all participants in both studies. Age group differences indicated worse cognitive function and vision in the older group compared to young (Table 1). Nonetheless, all participants had sufficient vision to undertake the obstacle crossing task.

**Table 1:** Basic demographic, anthropometric, and cognitive test performance information for part 1 (*n* = 49) and part 2 (*n* = 38) of the study

Sample characteristics	Part 1		*p* value	Part 2		*p* value
	Young group (*n* = 20)	Older group (*n* = 29)		Young group (*n* = 17)	Older group (*n* = 21)	
Descriptive						
Age (years)	25.5 (4.4)	74.2 (4.2)	*p* < 0.001	26.8 (4.5)	72.9 (4.5)	*p* < 0.001
Female (*n*)	8	18	0.128	9	11	0.973
Height (cm)	170 (8)	167 (10)	0.245	170 (9)	166 (9)	0.312
Weight (kg)	68 (14)	73 (18)	0.389	67 (10)	74 (13)	0.095
Corrected visual acuity (logMAR)	0.23 (0.09)	0.43 (0.15)	*p* < 0.001	0.22 (0.1)	0.41 (0.18)	*p* < 0.001
Cognitive						
MMSE score	29.7 (0.5)	29.0 (0.8)	0.002	29.8 (0.4)	29.2 (0.9)	0.018
TMT (B-A) (s)	20.3 (11.8)	47.8 (17.0)	*p* < 0.001	13.6 (9.7)	45.4 (19.8)	*p* < 0.001
Digit Span Backwards score	11.5 (2.6)	9.5 (2.5)	0.009	11.8 (2.4)	9.4 (2.6)	0.006
Visuospatial star movement test score	10.0 (0)	9.6 (0.8)	0.007	10.0 (0)	9.6 (0.5)	0.003

Data are presented as mean (standard deviation) unless otherwise specified. Higher scores in the tests of vision and TMT (B-A) indicate poorer performances. Higher scores in the MMSE (range 0–30), WAIS-III Digit Span Backwards subtest (range 0–16) and the visuospatial star movement test (range 1–10) indicate better performances

*MMSE* Mini-mental state examination, *TMT (B-A)* trail making test (part B–part A)

According to Table 1, the proportion of women was not significantly different between the young and older groups in Study 1 (40% vs. 62%, respectively) (Χ^2^ = 2.315, *p* = 0.128) or Study 2 (53% vs. 52%) (Χ^2^ = 0.001, *p* = 0.973). Overall, women comprised 53% of participants in Study 1 and 53% in Study 2.

### Missing data

Participants had missing data for a single trial in the following conditions for Study 1: low zero-delay (*n* = 1), high zero-delay (*n* = 2), low short delay (*n* = 1), low long delay (*n* = 3), high short delay (*n* = 1) and high long delay (*n* = 3). In addition, two trials were missing for one participant in condition low zero-delay and for another participant in condition high short delay. For Study 2, the number of participants who had a single trial missing is as follows: low zero-delay (*n* = 2), low non-spatial (*n* = 4), low visuospatial (*n* = 2), high zero-delay (*n* = 1), high non-spatial (*n* = 2), high visuospatial (*n* = 4). These were due to a fallen or obstructed foot marker. No adjustment was needed in the statistical analysis given all participants had a full dataset of mean and intra-subject variability of TC.

### Scaling of leg trajectories to obstacle height

In both studies, the leading and trailing leg trajectories were scaled to the obstacle height. At the zero-delay condition (control trials) of study 1, the mean maximal toe elevation for both the leading (*F*_1,46_ = 574.159, *p* < 0.001) and trailing legs (*F*_1,46_ = 125.366, *p* < 0.001) were increased when stepping over the high obstacle compared to the low obstacle (main effect of height) (Data presented in supplementary Table S1).

In Study 1, two young and two older participants contacted the high obstacle (18 cm) with the lead foot on a single occasion (never on first obstacle crossing). In study 2, one young participant contacted the short obstacle (12 cm) with the lead foot. These trials were discarded and repeated. Four of the five participants (three young, one older) who contacted the obstacle met the minimum requirements for visual acuity for fitness to drive [corrected visual acuity (0.3logmar and above)] (Latham et al. 2015).

### Study 1 findings

**Fig. 3:**
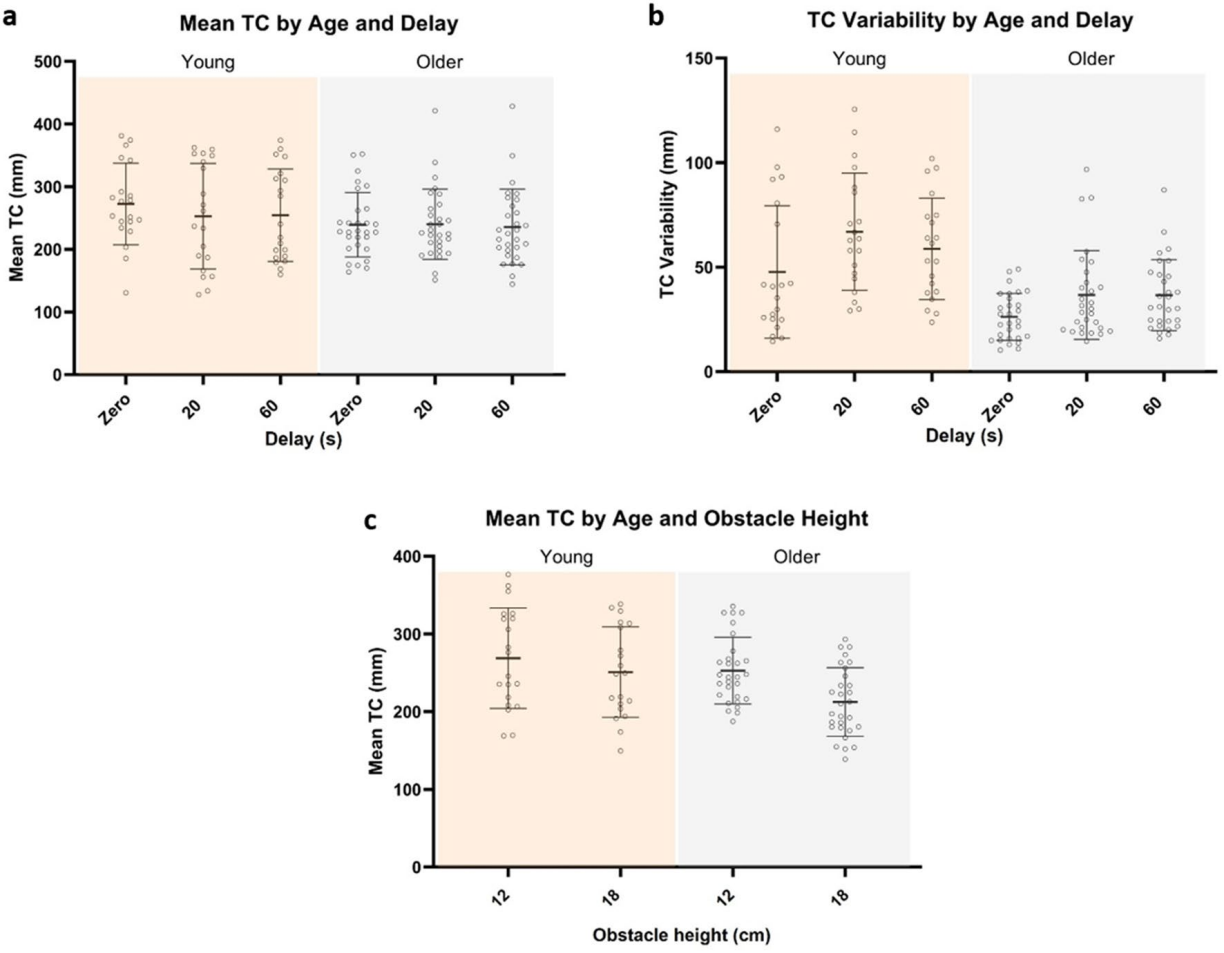
Study 1 **a** mean TC showed no age by delay interaction effect. **b** TC variability showed no age by delay interaction effect. **c** mean TC showed an age by height interaction effect. *Low* 12 cm obstacle height. *High* 18 cm obstacle height. Lines and error bars represent means and standard deviations

Contrary to our hypothesis, there were no age by delay interactions for the mean trailing leg TC (*F*_1,47_ = 0.567, *p* = 0.571) or intra-subject variability of the trailing leg TC (*F*_1,47_ = 1.982, *p* = 0.150) (Fig. 3a and b). Instead, for the mean trailing leg TC, we found a significant age group by obstacle height interaction (*F*_2,94_ = 13.838, *p* = 0.001) (Fig. 3c). Post hoc tests showed that both age groups had a significantly reduced mean trailing leg TC when stepping over the high obstacle compared to the low obstacle (young: *p* = 0.001, older: *p* < 0.001). In addition, when stepping over the low obstacle, there was no significant difference between the mean trailing leg TC used by both age groups (*p* = 0.621). However, when stepping over the high obstacle, older adults had a significantly smaller mean trailing leg TC than young adults (*p* = 0.044) (Fig. 3c).

There were no height by age group or height by delay interaction on the intra-subject variability of the trailing leg TC. With the data pooled across the two height conditions, we found that the intra-subject variability of the trailing TC showed a main effect of age group (*F*_1,47_ = 14.52, *p* < 0.001), as well as a main effect of delay condition (*F*_2,94_ = 17.54, *p* < 0.001) (Fig. 3b). For the main effect of age group, TC variability was significantly greater in young adults compared to older adults (Fig. 3b). For the main effect of delay condition, TC variability was significantly increased at both the 20 s (*p* < 0.001) and 60 s (*p* < 0.001) delay conditions when compared to the zero-delay condition (Fig. 3b).

### Study 2 findings

**Fig. 4:**
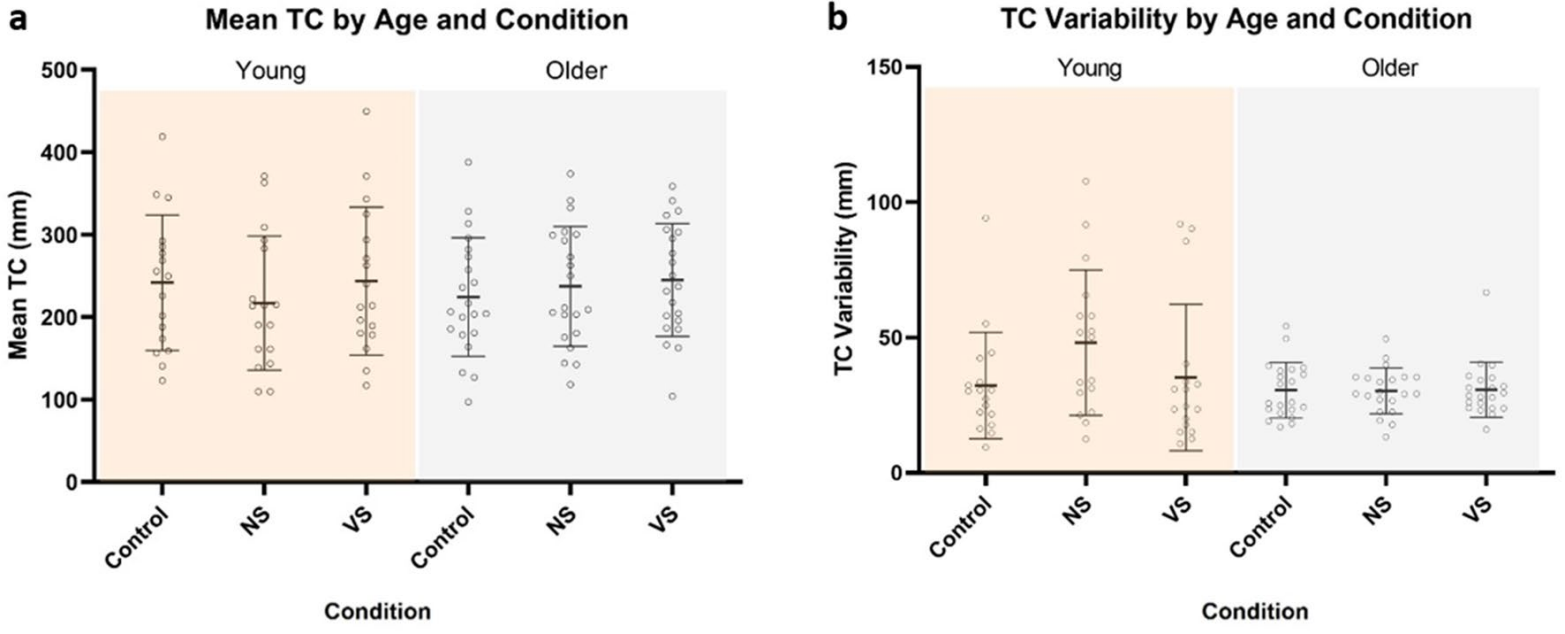
Study 2 **a** mean TC showed no age by condition interaction effect. **b** TC variability showed no age by condition interaction effect. Lines and error bars represent means and standard deviations. *NS* nonspatial. *VS* visuospatial

Contrary to our hypotheses, there were no age group by condition interactions for the mean trailing leg TC (*F*_2,72_ = 1.303, *p* = 0.278) or intra-subject variability of the trailing leg TC (*F*_2,72_ = 0.087, *p* = 0.917) (Fig. 4). There was also no age group by obstacle height interaction (*F*_2,72_ = 0.356, *p* = 0.554). The mean TC showed a main effect of obstacle height (*F*_1,36_ = 61.909, *p* < 0.001), where participants had a smaller mean TC when stepping over the high obstacle compared to the low obstacle. With data from both obstacle heights pooled, the mean TC showed no interaction effect (*F*_2,72_ = 1.303, *p* = 0.272) or main effect involving age group (*F*_2,72_ = 0.218, *p* = 0.643) or condition (*F*_2,72_ = 2.172, *p* = 0.140) (Fig. 4a).

There was no statistically significant height by age group or height by condition interaction for intra-subject variability of trailing leg TC. With the heights pooled, there was a main effect of condition (*F*_2,72_ = 3.59, *p* = 0.033), where participants showed greater TC variability when performing the NS secondary task compared to the control condition (i.e., no secondary task) (*p* = 0.021) (Fig. 4b). There was no difference in TC variability between the VS secondary task and control condition (*p* = 0.382), or the NS secondary task (*p* = 0.081). There was no main effect of age (*F*_1,36_ = 1.230, *p* = 0.275) (Fig. 4b).

All participants in both age groups recorded 100% accuracy in performing the visuospatial and arithmetic tasks.

## Discussion

### Summary

Our study 1 findings suggest that young and older adults alike were able to maintain an obstacle representation in VSWM for durations of at least 60 s, indicated by the subsequent ability to safely clear the obstacle with the trailing leg. However, the delay-dependent increase in trailing leg TC variability observed in both age groups suggests that this obstacle representation begins to deteriorate within the first 20 s. For study 2, our findings suggest that this VSWM-guided obstacle crossing performance is susceptible to dual-task interference, as indicated by the increase in TC variability observed in both age groups when a nonspatial (arithmetic) secondary task is performed compared to the control (single-task) condition. Interestingly, the absence of an age by condition interaction effect on dual-task obstacle crossing performance suggests that ageing does not increase the dual-task cost to trail foot clearance during obstacle crossing, at least at the level of task complexity investigated in this study.

### Study 1: maintaining an obstacle representation in visuospatial working memory

In study 1, we used an obstacle crossing paradigm to investigate the effects of age and delay duration on the storage of an obstacle representation in VSWM, which is used to guide subsequent obstacle crossing behaviour. The ability to safely step over an obstacle with adequate clearance is crucial to safe locomotion. This task relies strongly on various higher level cognitive processes including VSWM, where visuospatial information of an obstacle’s dimensions and location (Diaz, et al. 2018) is temporarily stored and used to guide the trajectory of both lower limbs to appropriately clear the obstacle (Baddeley 2003; Pasternak et al. 2005). For instance, previous studies have demonstrated that cats and healthy young adults alike are able to successfully avoid obstacles along a walkway even when vision is withdrawn a few steps prior to obstacle crossing (Mohagheghi et al. 2004; Wilkinson et al. 2005), indicating that precise visuospatial information of the obstacle’s dimensions and spatial position can be stored in VSWM for at least a short duration and used to guide appropriate foot trajectories over the obstacles.

In most cases of obstacle crossing, this obstacle representation only needs to be stored in VSWM for short durations, given that in humans the trailing leg step follows almost immediately after the leading leg step over the obstacle. However, in certain situations, the neural representation of an obstacle may need to be maintained for considerably longer durations to guide safe obstacle negotiation, such as remembering the position of a placed-down garden tool in the garden, a child’s toy in the living room, a pet on the porch and when navigating a cluttered hallway after switching off the lights. Using the obstacle-straddling paradigm (where participants cross an obstacle with their leading leg and straddle it for a delay duration before crossing it with their trailing leg), Lajoie and colleagues demonstrated that young adults are capable of storing a neural representation of an obstacle for durations of at least 120 s, as indicated by appropriate scaling of the trailing leg to the obstacle height following the delay (Lajoie et al. 2012).

In the present experiment, we extended this question to older adults, and showed that older adults could similarly maintain an obstacle representation in VSWM for delays of at least 60 s. This is indicated by their consistent ability to scale their trailing leg to the obstacle height from memory, even after the delay duration. Importantly, the finding that participants of both age groups scaled their leg trajectories to the obstacle height even after a delay duration suggests that participants did not merely rely on a default high-stepping strategy to cross the obstacle. Rather, they had gathered precise information of the obstacle’s height and position relative to their trailing foot and stored this information as a stable neural representation of the obstacle in VSWM and were able to successfully execute the step in proportion to this memory.

Interestingly, the absence of an interaction effect or main effect of delay duration on the mean trailing leg TC suggests that the obstacle representation remained stable in the VSWM of older adults for at least 60 s, which was the longest delay duration tested in this experiment. This finding is surprising, considering VSWM has been widely demonstrated to decline with age, both in the context of pen-and-paper tests of VSWM (Myerson et al. 1999; Vecchi et al. 2005; Bopp et al. 2007; Fiore et al. 2012) and in the functional setting of upper limb grasping accuracy (Cheng et al. 2012). It is possible that the 60 s delay duration used in this study was insufficient to reveal any potential age-related declines in VSWM for obstacle crossing, and that this age effect may become apparent with an increased range of delay durations tested. However, in this present study, we did not test delay durations of longer than 60 s for concerns of fatigue in the older participants.

### Study 1: The effect of age on intra-individual toe clearance variability

The finding that intra-subject trailing leg TC variability increased in both age groups when a delay duration was imposed is of interest, given that this measure is a strong indicator of tripping risk during obstacle crossing (Best et al. 2008). When crossing an obstacle, sufficient TC must be achieved to ensure that the obstacle is cleared safely. As such, an increased variability in TC when clearing an obstacle may increase the risk of obstacle contact, thereby increasing the risk of tripping and falling. In the present study, despite participants remaining able to clear the obstacle safely with their trailing leg following an extended delay period, the increased trailing leg TC variability between zero and 20 s delays seems to suggest that the obstacle representation stored in VSWM undergoes some degree of deterioration even within a period of 20 s, regardless of age group.

Contrary to our hypothesis, intra-subject trailing leg TC variability was greater in young adults than in older adults regardless of obstacle height or delay duration. This is contradictory to previous obstacle negotiation literature which have found no effects of age on trailing leg TC variability (Chen 1991; Muir et al. 2019). The finding that the younger group was more variable than the older group is intriguing, considering older adults demonstrate greater TC variability than young adults during level walking (Mills et al. 2008). It is possible that the older adults in our sample were limited by a greater biomechanical rigidity than the young adults (Boyer et al. 2017), and thus had higher consistency in generating the required motion to step over the obstacle with their trailing leg. Moreover, due to poorer balance control and poorer vision, the older participants may have been more attentive and careful in crossing the obstacle, whereas the younger group may have adopted a more nonchalant approach (Muir et al. 2020).

### Study 1: The effect of height on mean toe clearance

We also found that participants of both age groups stepped over the high obstacle with a smaller TC than the low obstacle, regardless of delay duration. This appears consistent with findings by Lajoie and colleagues (Lajoie et al. 2012) in young adults, although the obstacle heights used in their experiment were higher than those used here. Notably, previous obstacle negotiation studies have yielded inconsistent findings on the effect of obstacle height on mean TC, with some studies reporting a smaller TC with higher obstacles (Lajoie et al. 2012; Maidan et al. 2018), some reporting a larger TC with higher obstacles (Chen^1991^; Muir et al. 2019; Pan et al. 2016), and some reporting no effect of obstacle height on TC (Lu et al. 2006). There is also recent evidence in young adults that foot clearance of the lead and trail limbs are affected by the contralateral obstacle’s height in a dual-obstacle crossing paradigm (Miura et al. 2021). Nevertheless, the trend of decreasing TC with increasing obstacle height in this study is interesting as it is consistent with research showing that tripping risk increases with obstacle height (Chou et al. 1998; Rietdyk et al. 2011).

Interestingly, the tendency of decreasing trailing leg TC with increasing obstacle height was more evident in the older group, contrary to numerous previous studies which found no effect of age on trailing leg TC (Lu et al. 2006; Draganich et al. 2004; Lowrey et al. 2007; Chien, et al. 2018). A possible explanation for this observation may be the age-related differences in kinematic approach to obstacle crossing between young and older adults. For instance, McFadyen and Prince (McFadyen et al. 2002) found that older adults demonstrated greater pelvic drop ipsilateral to the trailing leg as it stepped over the obstacle, which contributed to a smaller TC when crossing an obstacle compared to young adults. Here, it is possible that these same age-related kinematic differences may have contributed to the smaller trailing leg TC seen in older adults compared to young adults when stepping over the high obstacle.

### Study 2: The effect of age in dual-task conditions

In study 2, we used a dual-task paradigm to investigate whether undertaking a VS secondary task as opposed to a non-VS task would interfere with VSWM necessary to guide obstacle crossing and subsequently impair obstacle clearance. Contrary to our hypothesis, we did not find an age by condition effect on obstacle crossing kinematic parameters (mean and intra-subject variability of trailing leg TC). This suggests that even when undertaking a cognitive secondary task, older adults remain as capable as young adults of storing a stable obstacle representation in VSWM for delay duration of at least 60 s.

A possible explanation for the absence of an age effect on dual-task interference for the VSWM-guided obstacle crossing task is that the older participants may have adopted a more cautious approach to obstacle crossing than their younger counterparts, choosing to prioritize performance in the obstacle crossing primary task to minimize tripping risk at the expense of secondary task performance and in the absence of specific instructions regarding task prioritization. This pattern of increasing motor task prioritization with age, often reported in dual-tasking literature (Kim et al. 2007; Muir et al. 2020; Brown et al. 2002; Schrodt et al. 2004; Mersmann et al. 2013; Simieli et al. 2015), may indicate an adaptive strategy by older adults to compensate for age-related declines in balance control which might otherwise have posed an increased fall risk, particularly in a situation requiring dual tasking (Sturnieks et al. 2008b).

An alternate explanation for our findings might be that the secondary cognitive tasks did not exceed the threshold of complexity required to challenge the attentional, working memory and executive function resources of our participants, which might otherwise have revealed age-related VSWM changes in our older participants. This explanation is supported by the finding that all participants in both age groups provided correct answers to all questions across both cognitive task types, suggesting a potential insufficient level of difficulty. Nonetheless, future studies should include recordings of response time, a more sensitive outcome measure of cognitive task performance.

### Study 2: Dual-task costs of the arithmetic and visuospatial tasks

The finding that the arithmetic secondary task increased the intra-subject trailing leg TC variability following a delay duration is concordant with findings from previous dual-tasking obstacle negotiation studies (Kim et al. 2007; Guadagnin et al. 2015). This increased variability in TC suggests that interference from the arithmetic task resulted in a poorer ability to consistently recall precise information about the obstacle height from VSWM to guide trailing leg obstacle clearance. Considering the failure to generate sufficient toe clearance accounts for the majority of obstacle-related trips (Chou et al. 1997), this finding implies that the concurrent performance of a cognitive task such as mental arithmetic during obstacle crossing may increase the risk of tripping from obstacle contact.

Surprisingly, the visuospatial task did not appear to interfere with the VSWM-guided obstacle crossing performance. This finding contrasts with those from previous dual-task studies wherein the same visuospatial star movement task was found to interfere with balance control during standing and stepping tasks more so than the difficulty-matched arithmetic task (Menant et al. 2014; Sturnieks et al. 2008b). In these studies, the disproportionally greater dual-task cost of the visuospatial task was attributed to its shared requirements for the same visuospatial processing neural pathways as the balance control tasks, leading to greater dual-task interference.

The current divergent findings may be due to experiment design differences, as unlike past studies that involved quick responses or dynamic movements, participants were standing in place over extended periods while undertaking the secondary tasks. As such, they would have time to use strategies to conduct the two cognitive tasks. In the case of the arithmetic task participants may have used the VSWM to store, manipulate, and update numerical information (Hubber et al. 2014; Cavdaroglu et al. 2016; Clearman et al. 2017), rather than complete the tasks by rote. Furthermore, under such unhurried and static conditions, it is possible some participants used fixtures in the laboratory as visual grids thereby reducing reliance on VSWM to perform the star movement task. Indeed, some participants volunteered reports of using this strategy at the completion of their experiment.

### Limitations

We acknowledge our study has certain limitations. In this experiment, participants began from a stationary position and took a single step toward the obstacle before crossing it with the leading leg. This method may have resulted in participants adopting different gait and obstacle crossing behaviours compared to that of natural gait and obstacle crossing. It is possible that knowledge about stopping versus continuing walking could have altered foot placement relative to the obstacle. We found significantly greater trail toe distance to the obstacle in the control trials versus the delay trials in both groups (data presented in supplementary Table S2). While this did not alter mean TC throughout the control and delay trials (Fig. 3a), we cannot rule out this factor may have contributed to the increased TC variability noted in the trials with ≥ 20 s delays (Fig. 3b). Moreover, participants may have paid additional attention to obstacle crossing performance in the experimental setting compared to what they would have in everyday life. Both these factors potentially limit the ecological validity of this experimental paradigm. Furthermore, this study only examined delay durations of up to 60 s, for concerns of fatigue in older adults as well as to reduce the time required for assessment of participants. However, it appears this delay duration was insufficient to reveal age-related differences in VSWM-guided obstacle crossing in our study sample. Thus, future studies investigating the effects of age on obstacle representation in VSWM could utilise longer delay durations to reveal a potential age effect on VSWM-guided obstacle crossing. In addition, in both studies, compared to the younger participants, older participants had significantly worse corrected visual acuity, which may have impaired their ability to gather accurate visual information to perform the obstacle crossing task and might have contributed to age group differences. In study 2, the verbal responses required by the secondary tasks introduced further motor demands in addition to the obstacle crossing primary task; this increased task complexity was not controlled for in the control trials. A majority of participants (12 young and 17 older people) performed both studies. However, there were no statistically significant differences in TC variability between those who did (*n* = 29) and those who did not (*n* = 9) take part in both studies, suggesting a learning effect between Study 1 and Study 2 is unlikely. Finally, we acknowledge that the computation of the variability measure (trail leg TC) for the control trials of Studies 1 and 2 as well as for the experimental trials of Study 2 was based on only six trials; while small, this number of repetitions was selected to minimise fatigue in the older group and monotony across both groups. Finally we acknowledge that the toe clearances we report here are high but still within the magnitude expected for this variable. By no means did this project aim to provide normative data for mean TC. As indicated in “Protocol”, our protocol was modelled on the obstacle-straddling paradigm described by Lajoie and colleagues (Lajoie et al. 2012). In the present study, we positioned our toe markers in the same location as described in previous papers which used similar protocols (Shinya et al. 2012; Lajoie et al. 2012).

## Conclusion

In conclusion, contrary to our hypotheses, our findings suggest that young and older adults alike can store an obstacle representation in VSWM for durations of at least 60 s and use this information to safely scale their trailing leg over an obstacle. However, the increase in trailing leg TC variability with delay duration suggests that this obstacle representation starts to deteriorate even within the first 20 s. The finding that undertaking a concurrent arithmetic task impaired VSWM-guided obstacle clearance suggests a potential increased risk of tripping during obstacle crossing while dual tasking in both young and older people.

## Supplementary Information

Below is the link to the electronic supplementary material.

Supplementary file1 (DOCX 14 KB)

Supplementary file2 (DOCX 13 KB)

## Data Availability

The datasets generated during and/or analysed during the current study are available from the corresponding author on reasonable request.
