# Supplementary material for: Visuospatial working memory and obstacle crossing in young and older people
Source: Exp Brain Res. 2022 Sep 16;240(11):2871–83. doi: 10.1007/s00221-022-06458-9 (PMC9587940; doi:10.1007/s00221-022-06458-9)
Supplement: Supplementary file 1 — Supplementary file1 (DOCX 14 KB) [file 221_2022_6458_MOESM1_ESM.docx]

Table S1. Mean (standard deviation) values for the mean trailing and leading leg maximal toe elevation (MTE) by age group, delay duration, and obstacle height.

|  | | **Zero delay** | | **20s delay** | | **60s delay** | |
| --- | --- | --- | --- | --- | --- | --- | --- |
|  |  | **Low height** | **High height** | **Low height** | **High height** | **Low height** | **High height** |
| **Mean trailing leg MTE** | **Young** | 361 (73) | 416 (70) | 354 (85) | 412 (88) | 364 (78) | 399 (82) |
|  | **Older** | 338 (50) | 377 (60) | 356 (56) | 374 (55) | 350 (61) | 373 (59) |
| **Mean leading leg MTE** | **Young** | 296 (41) | 358 (38) | 270 (40) | 325 (42) | 268 (40) | 330 (41) |
|  | **Older** | 310 (31) | 356 (32) | 275 (32) | 318 (32) | 275 (32) | 317 (31) |

All values are given in mm. MTE: mean toe elevation.
