# Supplementary material for: Visuospatial working memory and obstacle crossing in young and older people
Source: Exp Brain Res. 2022 Sep 16;240(11):2871–83. doi: 10.1007/s00221-022-06458-9 (PMC9587940; doi:10.1007/s00221-022-06458-9)
Supplement: Supplementary file 2 — Supplementary file2 (DOCX 13 KB) [file 221_2022_6458_MOESM2_ESM.docx]

**Table S2.** Trail toe marker distance to the obstacle prior to crossing; data are mean (SD).

|  | **Control trials** | **Delay trials** | **Mean difference (95% confidence intervals), p** |
| --- | --- | --- | --- |
| **Young (n=20)** | 187.4 (57.0) | 134.1 (49.2) | 50.8 (30.0 to 79.0), p<0.001 |
| **Old (n=28)** | 174.6 (36.1) | 115.2 (27.2) | 57.1 (43.0 to 71.2), p<0.001 |
| **All** | 179.9 (45.7) | 122.8 (38.4) | 56.0 (43.6 to 68.5), p<0.001 |

All values are given in mm.
